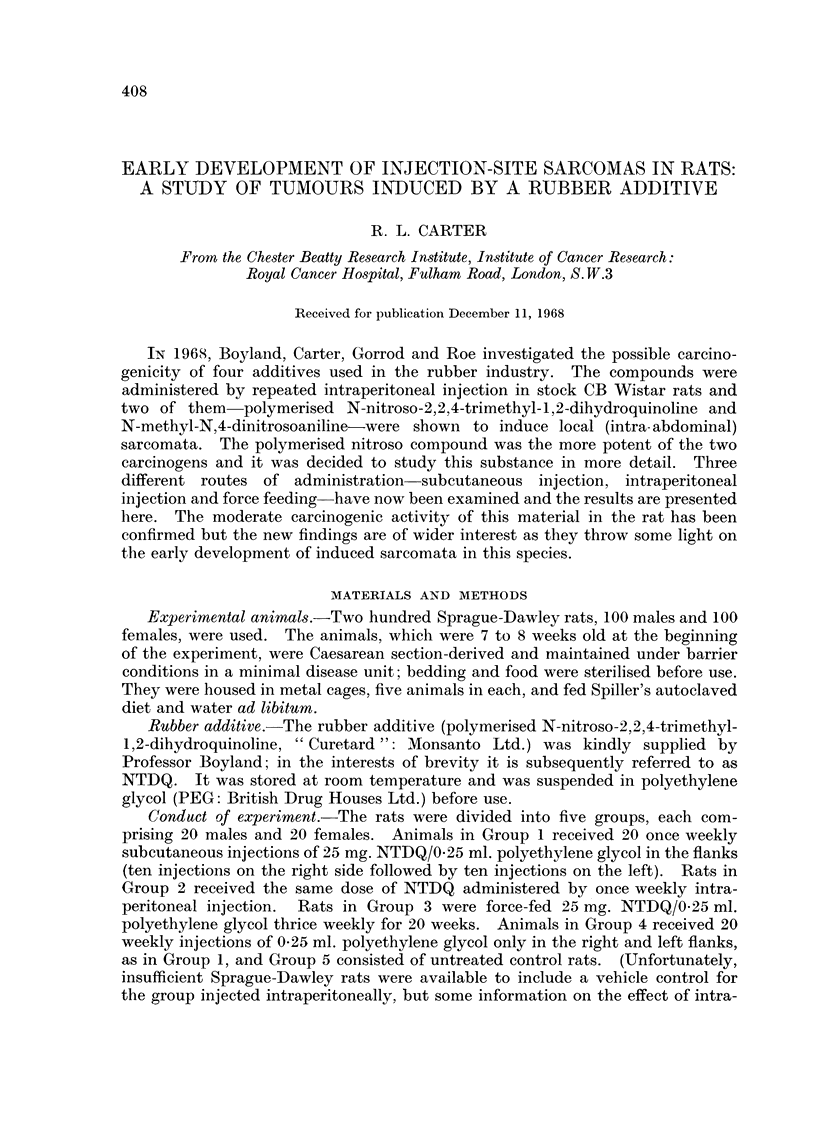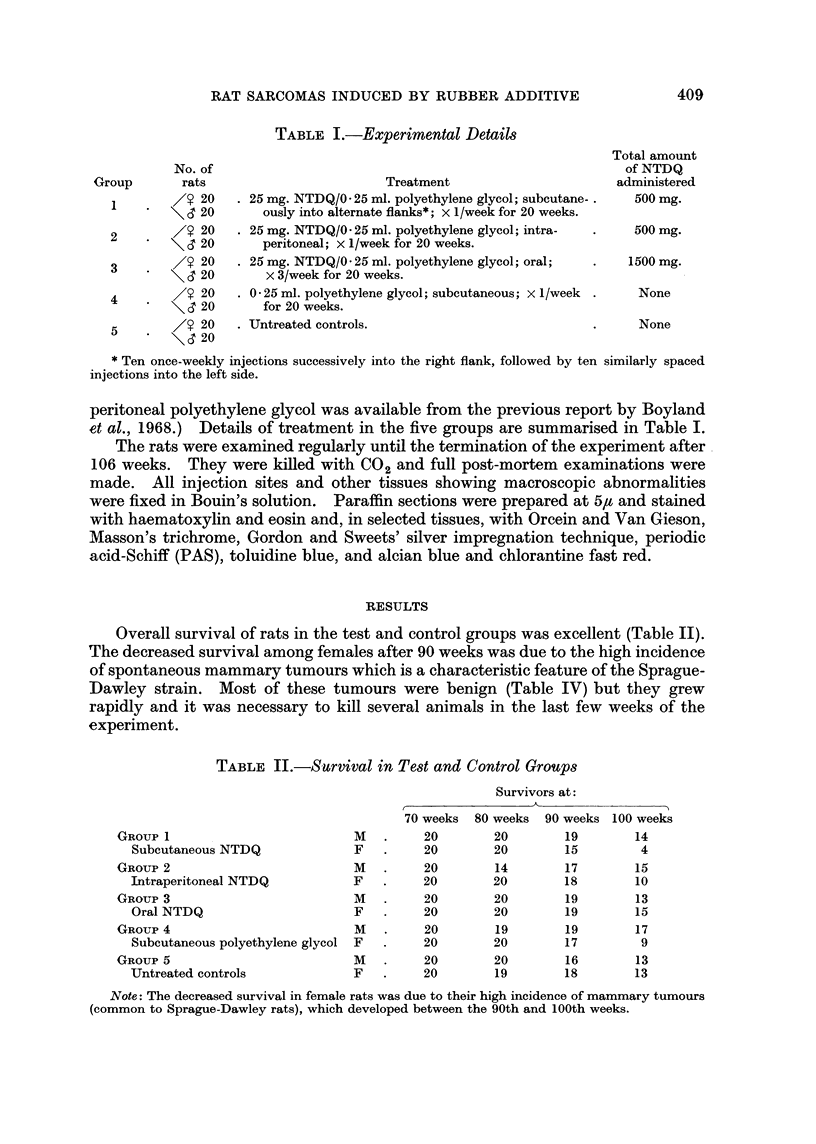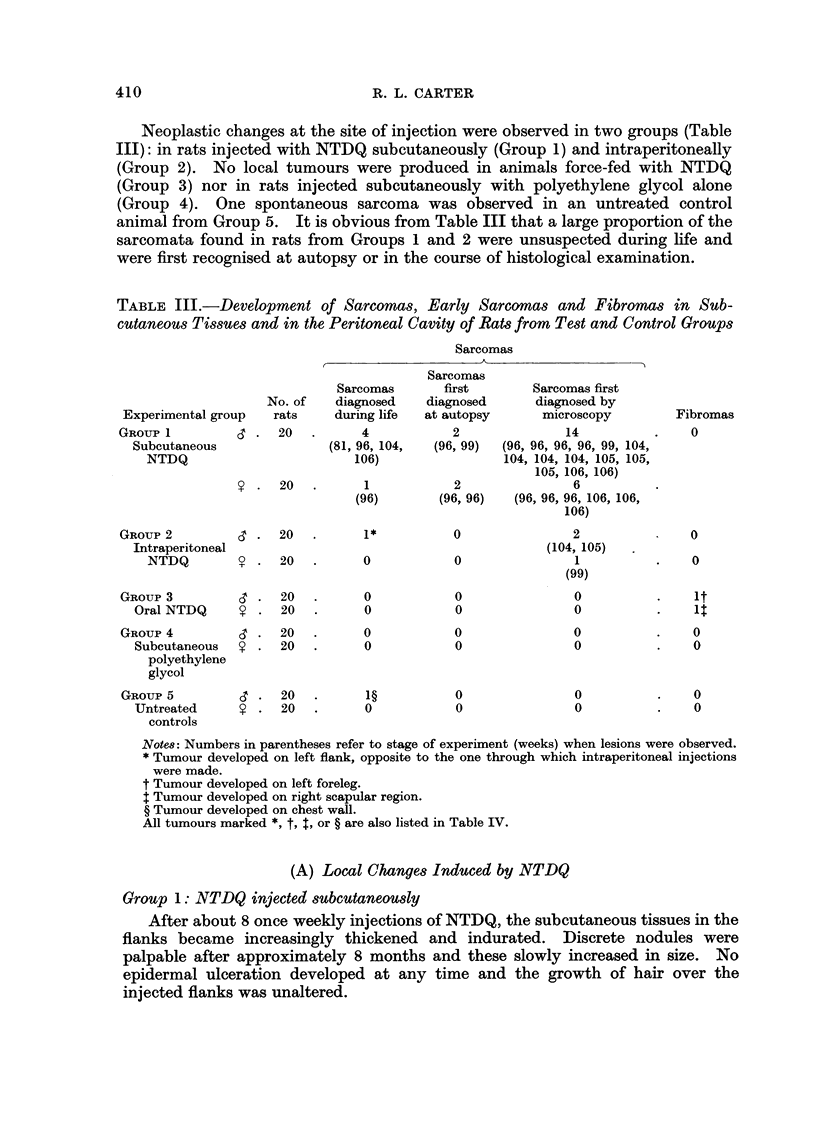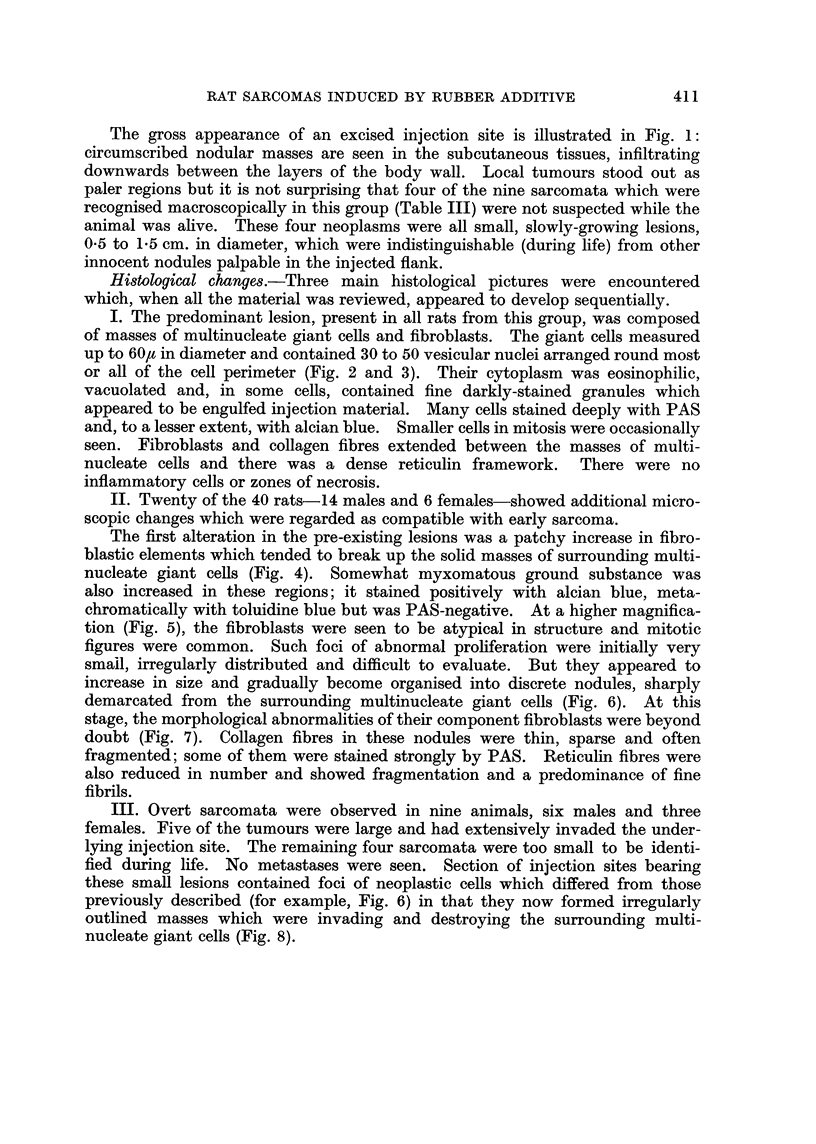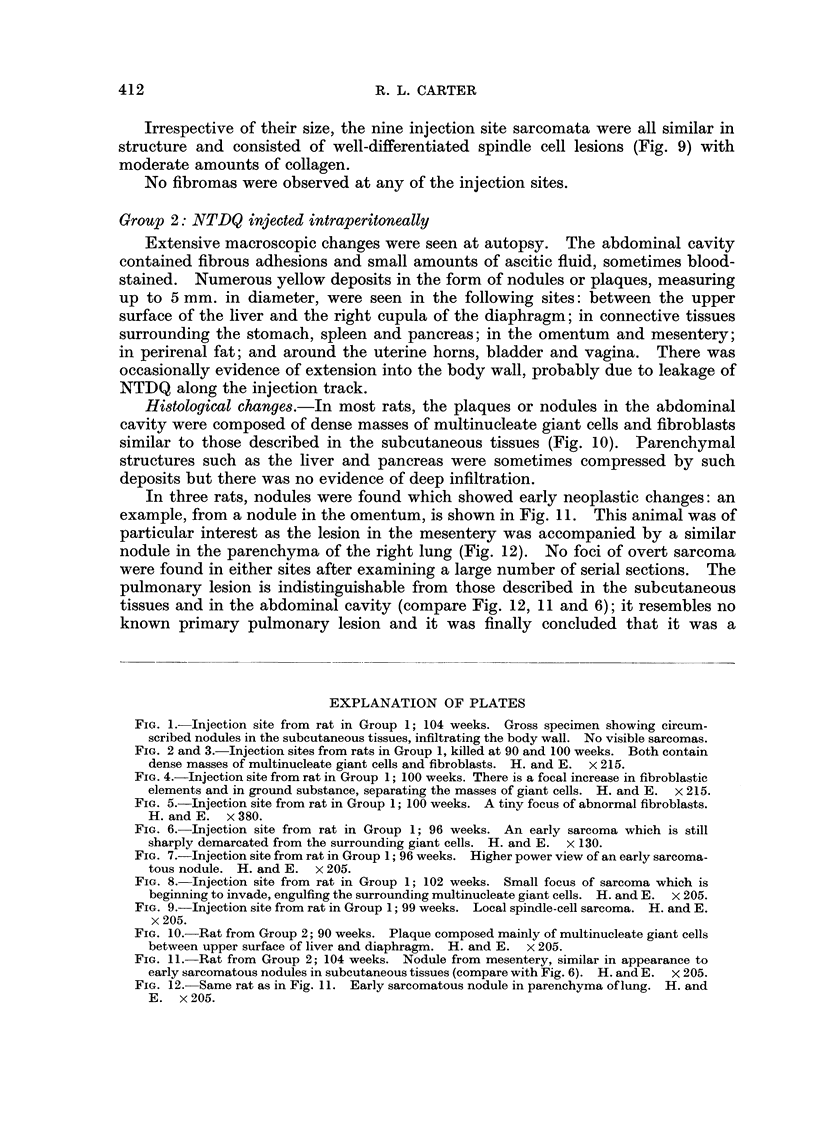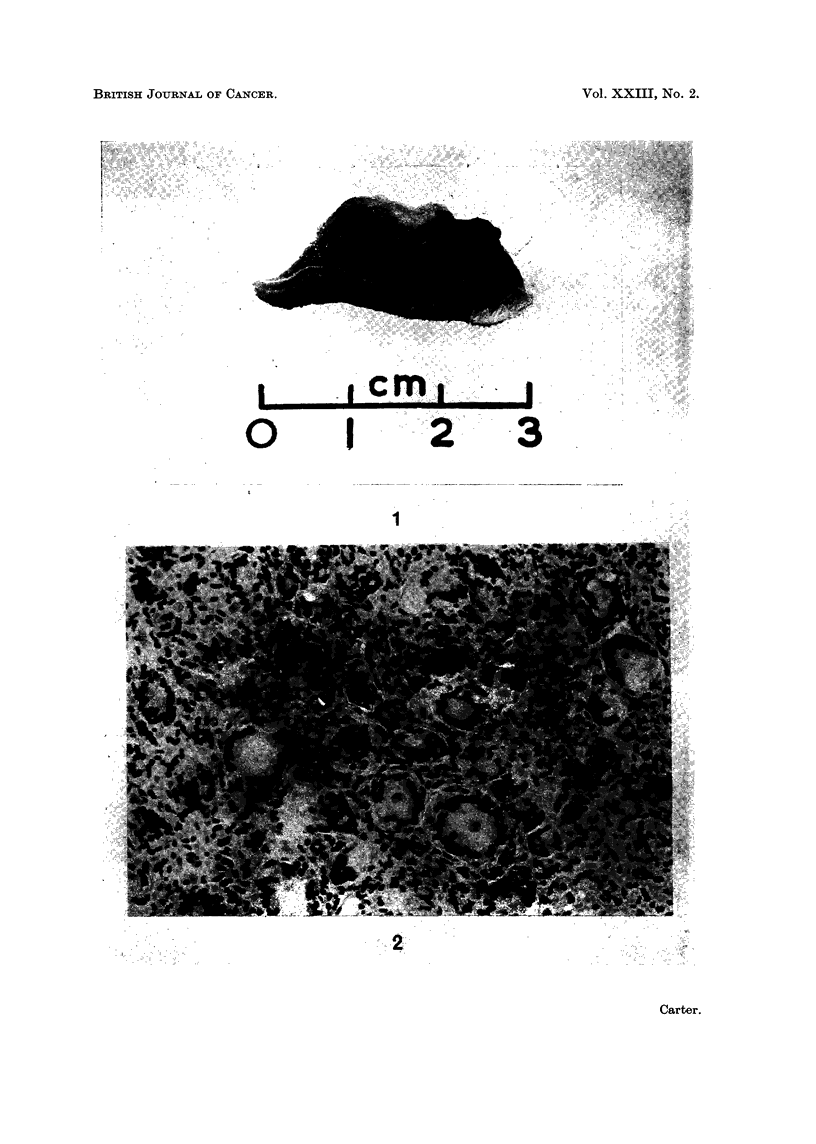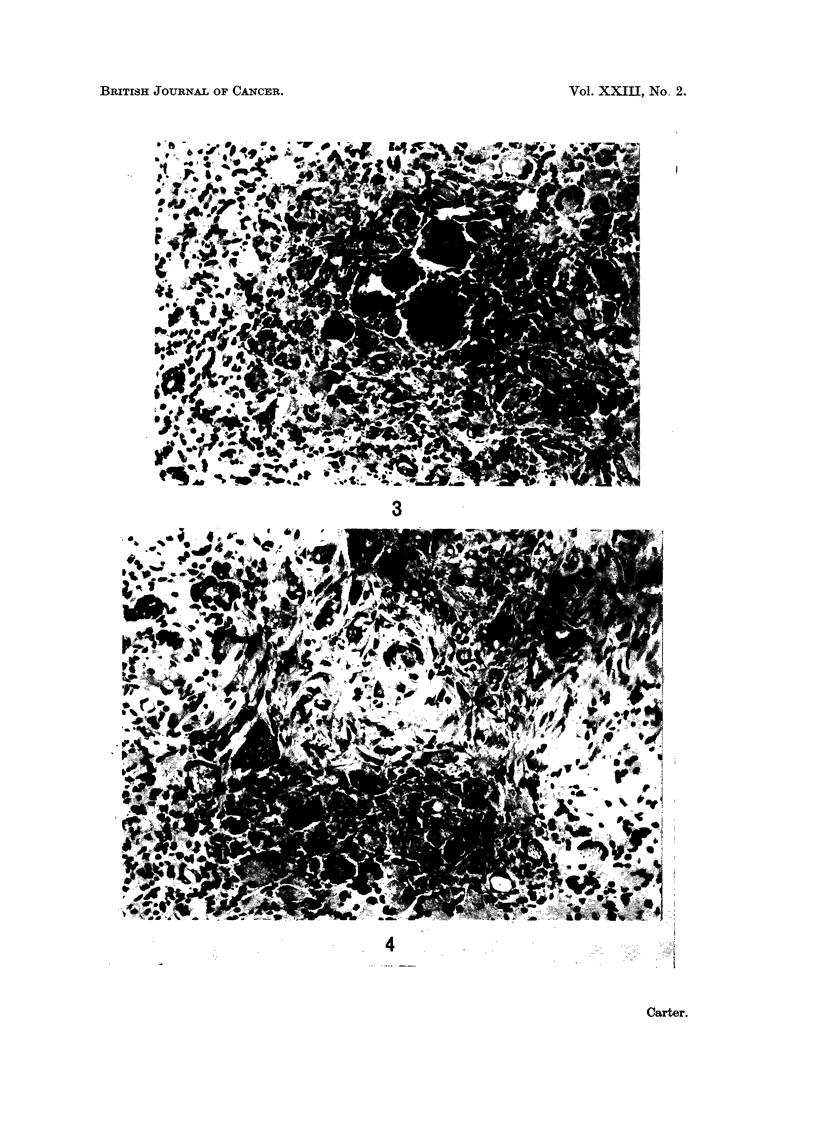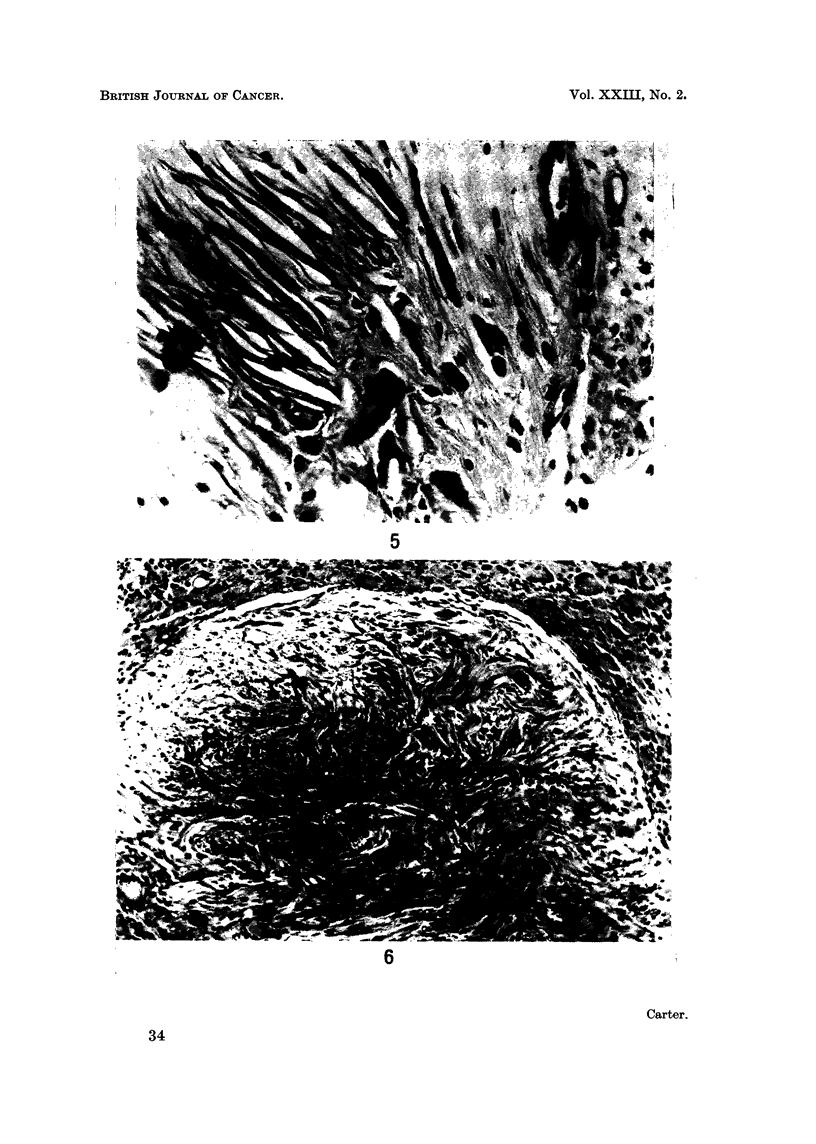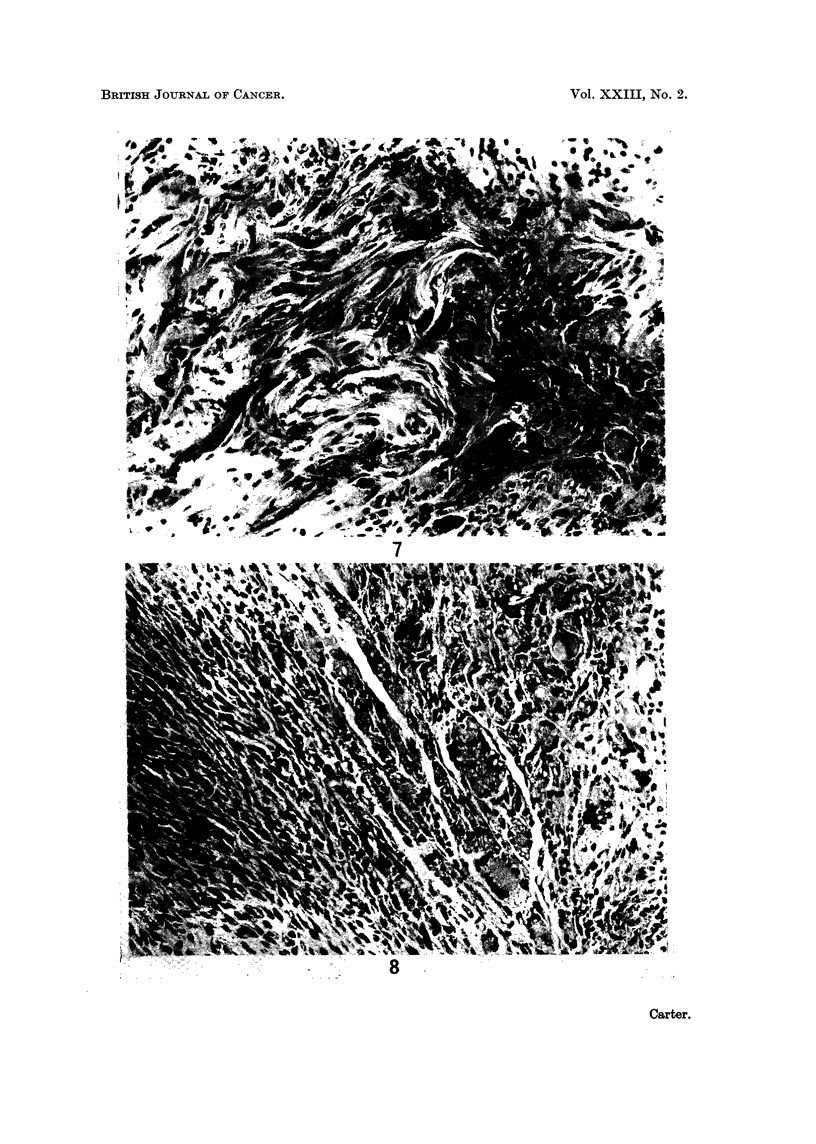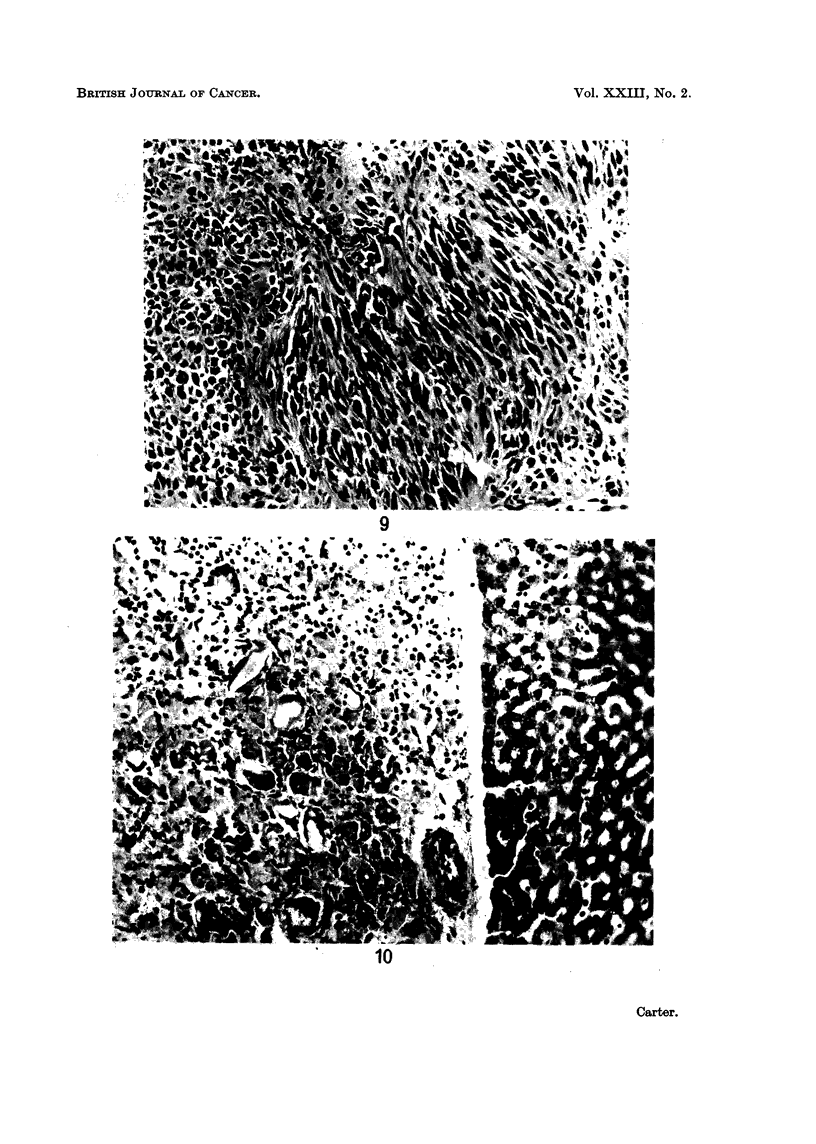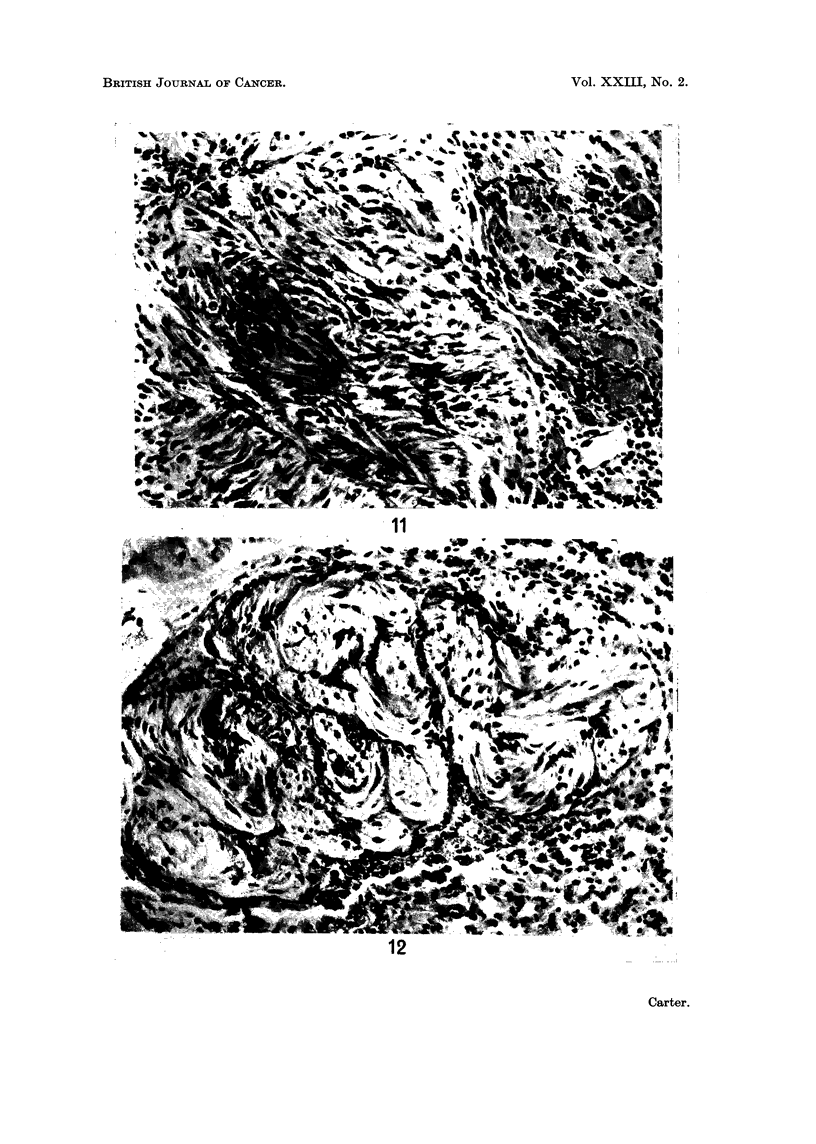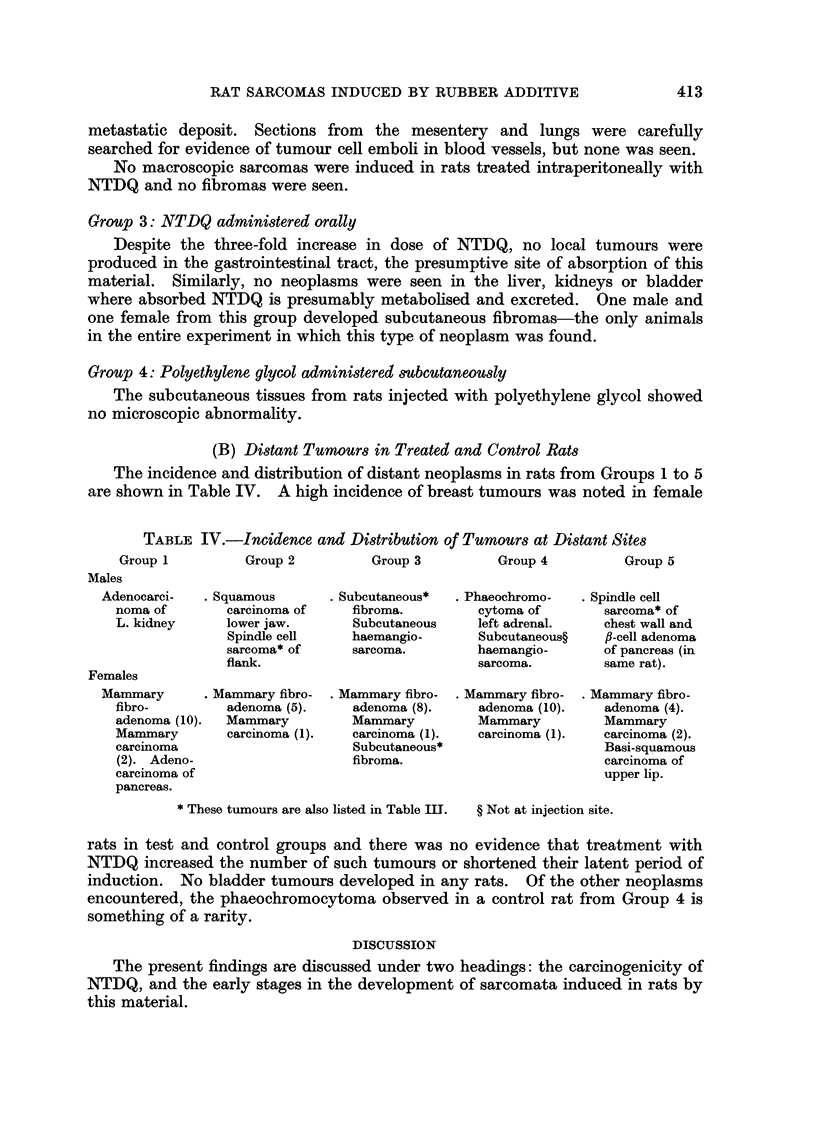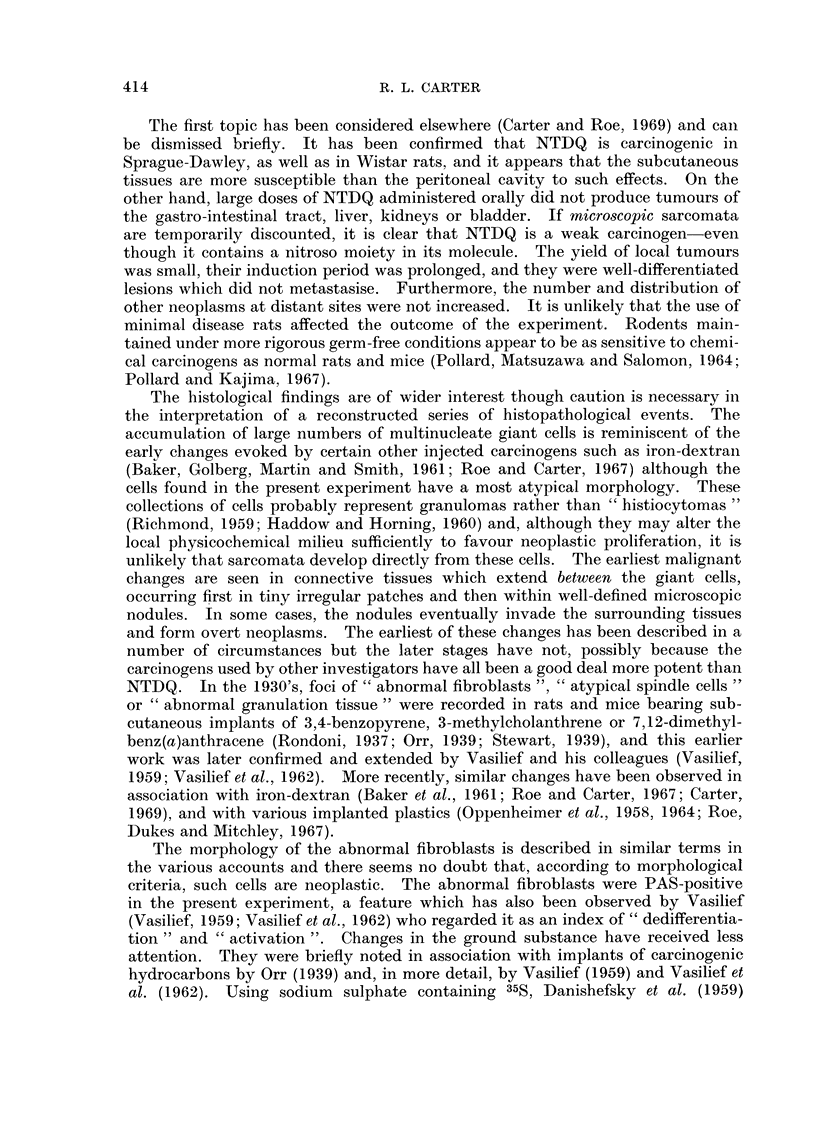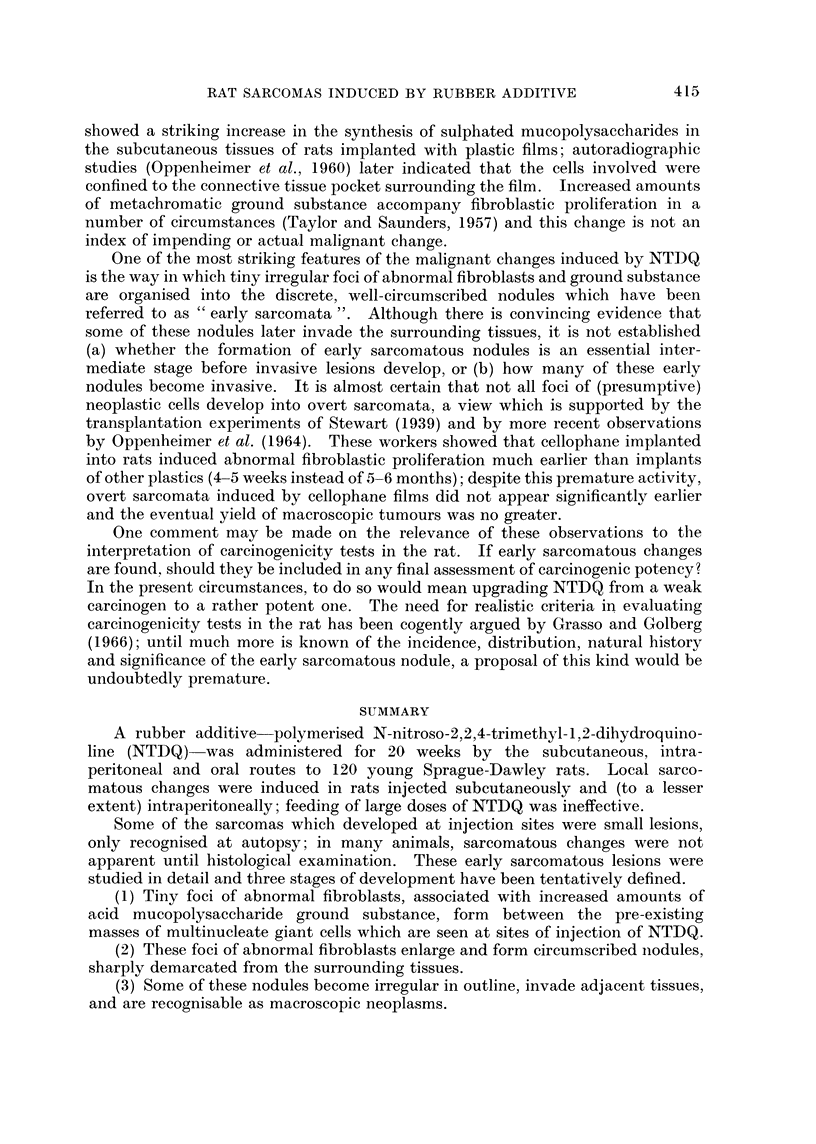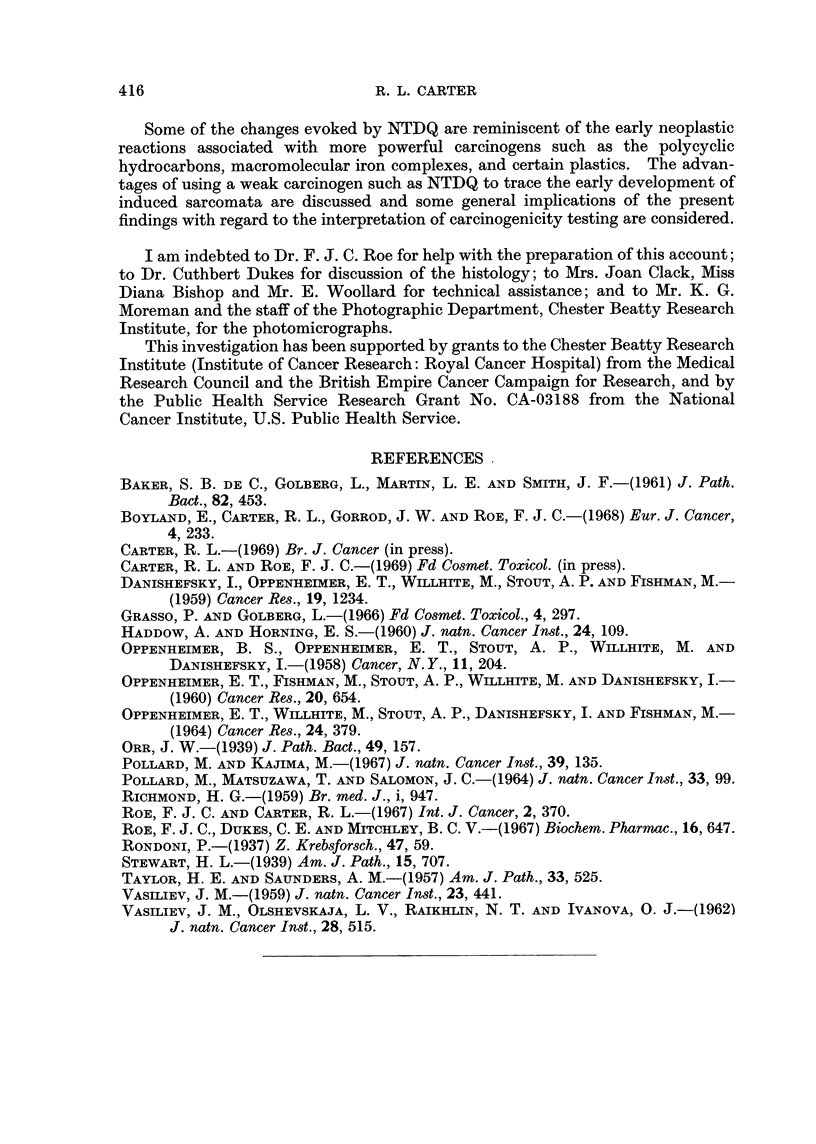# Early development of injection-site sarcomas in rats: a study of tumours induced by a rubber additive.

**DOI:** 10.1038/bjc.1969.53

**Published:** 1969-06

**Authors:** R. L. Carter

## Abstract

**Images:**


### EARLY DEVELOPMENT OF INJECTION-SITE SARCOMAS IN RATS: A STUDY OF TUMOURS INDUCED BY A RUBBER ADDITIVE

R. L. CARTER

*From the Chester Beatty Research Institute, Institute of Cancer Research: Royal Cancer Hospital, Fulham Road, London, S.W.3*

Received for publication December 11, 1968

In 1968, Boyland, Carter, Gorrod and Roe investigated the possible carcinogenicity of four additives used in the rubber industry. The compounds were administered by repeated intraperitoneal injection in stock CB Wistar rats and two of them—polymerised N-nitroso-2,2,4-trimethyl-1,2-dihydroquinoline and N-methyl-N,4-dinitrosoaniline—were shown to induce local (intra-abdominal) sarcomata. The polymerised nitroso compound was the more potent of the two carcinogens and it was decided to study this substance in more detail. Three different routes of administration—subcutaneous injection, intraperitoneal injection and force feeding—have now been examined and the results are presented here. The moderate carcinogenic activity of this material in the rat has been confirmed but the new findings are of wider interest as they throw some light on the early development of induced sarcomata in this species.

### MATERIALS AND METHODS

*Experimental animals.*—Two hundred Sprague-Dawley rats, 100 males and 100 females, were used. The animals, which were 7 to 8 weeks old at the beginning of the experiment, were Caesarean section-derived and maintained under barrier conditions in a minimal disease unit; bedding and food were sterilised before use. They were housed in metal cages, five animals in each, and fed Spiller's autoclaved diet and water *ad libitum.*

*Rubber additive.*—The rubber additive (polymerised N-nitroso-2,2,4-trimethyl-1,2-dihydroquinoline, "Curetard": Monsanto Ltd.) was kindly supplied by Professor Boyland; in the interests of brevity it is subsequently referred to as NTDQ. It was stored at room temperature and was suspended in polyethylene glycol (PEG: British Drug Houses Ltd.) before use.

*Conduct of experiment.*—The rats were divided into five groups, each comprising 20 males and 20 females. Animals in Group 1 received 20 once weekly subcutaneous injections of 25 mg. NTDQ/0·25 ml. polyethylene glycol in the flanks (ten injections on the right side followed by ten injections on the left). Rats in Group 2 received the same dose of NTDQ administered by once weekly intraperitoneal injection. Rats in Group 3 were force-fed 25 mg. NTDQ/0·25 ml. polyethylene glycol thrice weekly for 20 weeks. Animals in Group 4 received 20 weekly injections of 0·25 ml. polyethylene glycol only in the right and left flanks, as in Group 1, and Group 5 consisted of untreated control rats. (Unfortunately, insufficient Sprague-Dawley rats were available to include a vehicle control for the group injected intraperitoneally, but some information on the effect of intra-

TABLE I.—*Experimental Details*

| Group | No. of rats | Treatment | Total amount of NTDQ administered |
|---|---|---|---|
| 1 | ♀ 20<br>♂ 20 | 25 mg. NTDQ/0·25 ml. polyethylene glycol; subcutaneously into alternate flanks*; × 1/week for 20 weeks. | 500 mg. |
| 2 | ♀ 20<br>♂ 20 | 25 mg. NTDQ/0·25 ml. polyethylene glycol; intraperitoneal; × 1/week for 20 weeks. | 500 mg. |
| 3 | ♀ 20<br>♂ 20 | 25 mg. NTDQ/0·25 ml. polyethylene glycol; oral; × 3/week for 20 weeks. | 1500 mg. |
| 4 | ♀ 20<br>♂ 20 | 0·25 ml. polyethylene glycol; subcutaneous; × 1/week for 20 weeks. | None |
| 5 | ♀ 20<br>♂ 20 | Untreated controls. | None |

* Ten once-weekly injections successively into the right flank, followed by ten similarly spaced injections into the left side.

peritoneal polyethylene glycol was available from the previous report by Boyland *et al.*, 1968.) Details of treatment in the five groups are summarised in Table I.

The rats were examined regularly until the termination of the experiment after 106 weeks. They were killed with $CO_2$ and full post-mortem examinations were made. All injection sites and other tissues showing macroscopic abnormalities were fixed in Bouin's solution. Paraffin sections were prepared at 5$\mu$ and stained with haematoxylin and eosin and, in selected tissues, with Orcein and Van Gieson, Masson's trichrome, Gordon and Sweets' silver impregnation technique, periodic acid-Schiff (PAS), toluidine blue, and alcian blue and chlorantine fast red.

### RESULTS

Overall survival of rats in the test and control groups was excellent (Table II). The decreased survival among females after 90 weeks was due to the high incidence of spontaneous mammary tumours which is a characteristic feature of the Sprague-Dawley strain. Most of these tumours were benign (Table IV) but they grew rapidly and it was necessary to kill several animals in the last few weeks of the experiment.

TABLE II.—*Survival in Test and Control Groups*

| | | Survivors at: | | | |
|---|---|---|---|---|---|
| | | 70 weeks | 80 weeks | 90 weeks | 100 weeks |
| GROUP 1 | M | 20 | 20 | 19 | 14 |
| Subcutaneous NTDQ | F | 20 | 20 | 15 | 4 |
| GROUP 2 | M | 20 | 14 | 17 | 15 |
| Intraperitoneal NTDQ | F | 20 | 20 | 18 | 10 |
| GROUP 3 | M | 20 | 20 | 19 | 13 |
| Oral NTDQ | F | 20 | 20 | 19 | 15 |
| GROUP 4 | M | 20 | 19 | 19 | 17 |
| Subcutaneous polyethylene glycol | F | 20 | 20 | 17 | 9 |
| GROUP 5 | M | 20 | 20 | 16 | 13 |
| Untreated controls | F | 20 | 19 | 18 | 13 |

*Note*: The decreased survival in female rats was due to their high incidence of mammary tumours (common to Sprague-Dawley rats), which developed between the 90th and 100th weeks.

Neoplastic changes at the site of injection were observed in two groups (Table III): in rats injected with NTDQ subcutaneously (Group 1) and intraperitoneally (Group 2). No local tumours were produced in animals force-fed with NTDQ (Group 3) nor in rats injected subcutaneously with polyethylene glycol alone (Group 4). One spontaneous sarcoma was observed in an untreated control animal from Group 5. It is obvious from Table III that a large proportion of the sarcomata found in rats from Groups 1 and 2 were unsuspected during life and were first recognised at autopsy or in the course of histological examination.

TABLE III.—*Development of Sarcomas, Early Sarcomas and Fibromas in Subcutaneous Tissues and in the Peritoneal Cavity of Rats from Test and Control Groups*

| Experimental group | | No. of rats | Sarcomas: Sarcomas diagnosed during life | Sarcomas: Sarcomas first diagnosed at autopsy | Sarcomas: Sarcomas first diagnosed by microscopy | Fibromas |
|---|---|---|---|---|---|---|
| GROUP 1 Subcutaneous NTDQ | ♂ | 20 | 4 (81, 96, 104, 106) | 2 (96, 99) | 14 (96, 96, 96, 96, 99, 104, 104, 104, 104, 105, 105, 105, 106, 106) | 0 |
| | ♀ | 20 | 1 (96) | 2 (96, 96) | 6 (96, 96, 96, 106, 106, 106) | |
| GROUP 2 Intraperitoneal NTDQ | ♂ | 20 | 1* | 0 | 2 (104, 105) | 0 |
| | ♀ | 20 | 0 | 0 | 1 (99) | 0 |
| GROUP 3 Oral NTDQ | ♂ | 20 | 0 | 0 | 0 | 1† |
| | ♀ | 20 | 0 | 0 | 0 | 1‡ |
| GROUP 4 Subcutaneous polyethylene glycol | ♂ | 20 | 0 | 0 | 0 | 0 |
| | ♀ | 20 | 0 | 0 | 0 | 0 |
| GROUP 5 Untreated controls | ♂ | 20 | 1§ | 0 | 0 | 0 |
| | ♀ | 20 | 0 | 0 | 0 | 0 |

*Notes*: Numbers in parentheses refer to stage of experiment (weeks) when lesions were observed.
* Tumour developed on left flank, opposite to the one through which intraperitoneal injections were made.
† Tumour developed on left foreleg.
‡ Tumour developed on right scapular region.
§ Tumour developed on chest wall.
All tumours marked *, †, ‡, or § are also listed in Table IV.

### (A) *Local Changes Induced by NTDQ*

*Group 1: NTDQ injected subcutaneously*

After about 8 once weekly injections of NTDQ, the subcutaneous tissues in the flanks became increasingly thickened and indurated. Discrete nodules were palpable after approximately 8 months and these slowly increased in size. No epidermal ulceration developed at any time and the growth of hair over the injected flanks was unaltered.

The gross appearance of an excised injection site is illustrated in Fig. 1: circumscribed nodular masses are seen in the subcutaneous tissues, infiltrating downwards between the layers of the body wall. Local tumours stood out as paler regions but it is not surprising that four of the nine sarcomata which were recognised macroscopically in this group (Table III) were not suspected while the animal was alive. These four neoplasms were all small, slowly-growing lesions, 0·5 to 1·5 cm. in diameter, which were indistinguishable (during life) from other innocent nodules palpable in the injected flank.

*Histological changes.*—Three main histological pictures were encountered which, when all the material was reviewed, appeared to develop sequentially.

I. The predominant lesion, present in all rats from this group, was composed of masses of multinucleate giant cells and fibroblasts. The giant cells measured up to $60\mu$ in diameter and contained 30 to 50 vesicular nuclei arranged round most or all of the cell perimeter (Fig. 2 and 3). Their cytoplasm was eosinophilic, vacuolated and, in some cells, contained fine darkly-stained granules which appeared to be engulfed injection material. Many cells stained deeply with PAS and, to a lesser extent, with alcian blue. Smaller cells in mitosis were occasionally seen. Fibroblasts and collagen fibres extended between the masses of multinucleate cells and there was a dense reticulin framework. There were no inflammatory cells or zones of necrosis.

II. Twenty of the 40 rats—14 males and 6 females—showed additional microscopic changes which were regarded as compatible with early sarcoma.

The first alteration in the pre-existing lesions was a patchy increase in fibroblastic elements which tended to break up the solid masses of surrounding multinucleate giant cells (Fig. 4). Somewhat myxomatous ground substance was also increased in these regions; it stained positively with alcian blue, metachromatically with toluidine blue but was PAS-negative. At a higher magnification (Fig. 5), the fibroblasts were seen to be atypical in structure and mitotic figures were common. Such foci of abnormal proliferation were initially very small, irregularly distributed and difficult to evaluate. But they appeared to increase in size and gradually become organised into discrete nodules, sharply demarcated from the surrounding multinucleate giant cells (Fig. 6). At this stage, the morphological abnormalities of their component fibroblasts were beyond doubt (Fig. 7). Collagen fibres in these nodules were thin, sparse and often fragmented; some of them were stained strongly by PAS. Reticulin fibres were also reduced in number and showed fragmentation and a predominance of fine fibrils.

III. Overt sarcomata were observed in nine animals, six males and three females. Five of the tumours were large and had extensively invaded the underlying injection site. The remaining four sarcomata were too small to be identified during life. No metastases were seen. Section of injection sites bearing these small lesions contained foci of neoplastic cells which differed from those previously described (for example, Fig. 6) in that they now formed irregularly outlined masses which were invading and destroying the surrounding multinucleate giant cells (Fig. 8).

Irrespective of their size, the nine injection site sarcomata were all similar in structure and consisted of well-differentiated spindle cell lesions (Fig. 9) with moderate amounts of collagen.

No fibromas were observed at any of the injection sites.

*Group 2: NTDQ injected intraperitoneally*

Extensive macroscopic changes were seen at autopsy. The abdominal cavity contained fibrous adhesions and small amounts of ascitic fluid, sometimes blood-stained. Numerous yellow deposits in the form of nodules or plaques, measuring up to 5 mm. in diameter, were seen in the following sites: between the upper surface of the liver and the right cupula of the diaphragm; in connective tissues surrounding the stomach, spleen and pancreas; in the omentum and mesentery; in perirenal fat; and around the uterine horns, bladder and vagina. There was occasionally evidence of extension into the body wall, probably due to leakage of NTDQ along the injection track.

*Histological changes.*—In most rats, the plaques or nodules in the abdominal cavity were composed of dense masses of multinucleate giant cells and fibroblasts similar to those described in the subcutaneous tissues (Fig. 10). Parenchymal structures such as the liver and pancreas were sometimes compressed by such deposits but there was no evidence of deep infiltration.

In three rats, nodules were found which showed early neoplastic changes: an example, from a nodule in the omentum, is shown in Fig. 11. This animal was of particular interest as the lesion in the mesentery was accompanied by a similar nodule in the parenchyma of the right lung (Fig. 12). No foci of overt sarcoma were found in either sites after examining a large number of serial sections. The pulmonary lesion is indistinguishable from those described in the subcutaneous tissues and in the abdominal cavity (compare Fig. 12, 11 and 6); it resembles no known primary pulmonary lesion and it was finally concluded that it was a

---

EXPLANATION OF PLATES

FIG. 1.—Injection site from rat in Group 1; 104 weeks. Gross specimen showing circumscribed nodules in the subcutaneous tissues, infiltrating the body wall. No visible sarcomas.

FIG. 2 and 3.—Injection sites from rats in Group 1, killed at 90 and 100 weeks. Both contain dense masses of multinucleate giant cells and fibroblasts. H. and E. ×215.

FIG. 4.—Injection site from rat in Group 1; 100 weeks. There is a focal increase in fibroblastic elements and in ground substance, separating the masses of giant cells. H. and E. ×215.

FIG. 5.—Injection site from rat in Group 1; 100 weeks. A tiny focus of abnormal fibroblasts. H. and E. ×380.

FIG. 6.—Injection site from rat in Group 1; 96 weeks. An early sarcoma which is still sharply demarcated from the surrounding giant cells. H. and E. ×130.

FIG. 7.—Injection site from rat in Group 1; 96 weeks. Higher power view of an early sarcomatous nodule. H. and E. ×205.

FIG. 8.—Injection site from rat in Group 1; 102 weeks. Small focus of sarcoma which is beginning to invade, engulfing the surrounding multinucleate giant cells. H. and E. ×205.

FIG. 9.—Injection site from rat in Group 1; 99 weeks. Local spindle-cell sarcoma. H. and E. ×205.

FIG. 10.—Rat from Group 2; 90 weeks. Plaque composed mainly of multinucleate giant cells between upper surface of liver and diaphragm. H. and E. ×205.

FIG. 11.—Rat from Group 2; 104 weeks. Nodule from mesentery, similar in appearance to early sarcomatous nodules in subcutaneous tissues (compare with Fig. 6). H. and E. ×205.

FIG. 12.—Same rat as in Fig. 11. Early sarcomatous nodule in parenchyma of lung. H. and E. ×205.

1

2

3

4

Carter.

5

6

Carter.

7

8

Carter.

9

10

Carter.

11

12

Carter.

metastatic deposit. Sections from the mesentery and lungs were carefully searched for evidence of tumour cell emboli in blood vessels, but none was seen.

No macroscopic sarcomas were induced in rats treated intraperitoneally with NTDQ and no fibromas were seen.

*Group 3: NTDQ administered orally*

Despite the three-fold increase in dose of NTDQ, no local tumours were produced in the gastrointestinal tract, the presumptive site of absorption of this material. Similarly, no neoplasms were seen in the liver, kidneys or bladder where absorbed NTDQ is presumably metabolised and excreted. One male and one female from this group developed subcutaneous fibromas—the only animals in the entire experiment in which this type of neoplasm was found.

*Group 4: Polyethylene glycol administered subcutaneously*

The subcutaneous tissues from rats injected with polyethylene glycol showed no microscopic abnormality.

### (B) *Distant Tumours in Treated and Control Rats*

The incidence and distribution of distant neoplasms in rats from Groups 1 to 5 are shown in Table IV. A high incidence of breast tumours was noted in female

TABLE IV.—*Incidence and Distribution of Tumours at Distant Sites*

| | Group 1 | Group 2 | Group 3 | Group 4 | Group 5 |
|---|---|---|---|---|---|
| Males | Adenocarcinoma of L. kidney | Squamous carcinoma of lower jaw. Spindle cell sarcoma* of flank. | Subcutaneous* fibroma. Subcutaneous haemangio-sarcoma. | Phaeochromocytoma of left adrenal. Subcutaneous§ haemangio-sarcoma. | Spindle cell sarcoma* of chest wall and $\beta$-cell adenoma of pancreas (in same rat). |
| Females | Mammary fibro-adenoma (10). Mammary carcinoma (2). Adeno-carcinoma of pancreas. | Mammary fibro-adenoma (5). Mammary carcinoma (1). | Mammary fibro-adenoma (8). Mammary carcinoma (1). Subcutaneous* fibroma. | Mammary fibro-adenoma (10). Mammary carcinoma (1). | Mammary fibro-adenoma (4). Mammary carcinoma (2). Basi-squamous carcinoma of upper lip. |

* These tumours are also listed in Table III. § Not at injection site.

rats in test and control groups and there was no evidence that treatment with NTDQ increased the number of such tumours or shortened their latent period of induction. No bladder tumours developed in any rats. Of the other neoplasms encountered, the phaeochromocytoma observed in a control rat from Group 4 is something of a rarity.

### DISCUSSION

The present findings are discussed under two headings: the carcinogenicity of NTDQ, and the early stages in the development of sarcomata induced in rats by this material.

The first topic has been considered elsewhere (Carter and Roe, 1969) and can be dismissed briefly. It has been confirmed that NTDQ is carcinogenic in Sprague-Dawley, as well as in Wistar rats, and it appears that the subcutaneous tissues are more susceptible than the peritoneal cavity to such effects. On the other hand, large doses of NTDQ administered orally did not produce tumours of the gastro-intestinal tract, liver, kidneys or bladder. If *microscopic* sarcomata are temporarily discounted, it is clear that NTDQ is a weak carcinogen—even though it contains a nitroso moiety in its molecule. The yield of local tumours was small, their induction period was prolonged, and they were well-differentiated lesions which did not metastasise. Furthermore, the number and distribution of other neoplasms at distant sites were not increased. It is unlikely that the use of minimal disease rats affected the outcome of the experiment. Rodents maintained under more rigorous germ-free conditions appear to be as sensitive to chemical carcinogens as normal rats and mice (Pollard, Matsuzawa and Salomon, 1964; Pollard and Kajima, 1967).

The histological findings are of wider interest though caution is necessary in the interpretation of a reconstructed series of histopathological events. The accumulation of large numbers of multinucleate giant cells is reminiscent of the early changes evoked by certain other injected carcinogens such as iron-dextran (Baker, Golberg, Martin and Smith, 1961; Roe and Carter, 1967) although the cells found in the present experiment have a most atypical morphology. These collections of cells probably represent granulomas rather than " histiocytomas " (Richmond, 1959; Haddow and Horning, 1960) and, although they may alter the local physicochemical milieu sufficiently to favour neoplastic proliferation, it is unlikely that sarcomata develop directly from these cells. The earliest malignant changes are seen in connective tissues which extend *between* the giant cells, occurring first in tiny irregular patches and then within well-defined microscopic nodules. In some cases, the nodules eventually invade the surrounding tissues and form overt neoplasms. The earliest of these changes has been described in a number of circumstances but the later stages have not, possibly because the carcinogens used by other investigators have all been a good deal more potent than NTDQ. In the 1930's, foci of " abnormal fibroblasts ", " atypical spindle cells " or " abnormal granulation tissue " were recorded in rats and mice bearing subcutaneous implants of 3,4-benzopyrene, 3-methylcholanthrene or 7,12-dimethylbenz(*a*)anthracene (Rondoni, 1937; Orr, 1939; Stewart, 1939), and this earlier work was later confirmed and extended by Vasilief and his colleagues (Vasilief, 1959; Vasilief *et al.*, 1962). More recently, similar changes have been observed in association with iron-dextran (Baker *et al.*, 1961; Roe and Carter, 1967; Carter, 1969), and with various implanted plastics (Oppenheimer *et al.*, 1958, 1964; Roe, Dukes and Mitchley, 1967).

The morphology of the abnormal fibroblasts is described in similar terms in the various accounts and there seems no doubt that, according to morphological criteria, such cells are neoplastic. The abnormal fibroblasts were PAS-positive in the present experiment, a feature which has also been observed by Vasilief (Vasilief, 1959; Vasilief *et al.*, 1962) who regarded it as an index of " dedifferentiation " and " activation ". Changes in the ground substance have received less attention. They were briefly noted in association with implants of carcinogenic hydrocarbons by Orr (1939) and, in more detail, by Vasilief (1959) and Vasilief *et al.* (1962). Using sodium sulphate containing $^{35}S$, Danishefsky *et al.* (1959)

showed a striking increase in the synthesis of sulphated mucopolysaccharides in the subcutaneous tissues of rats implanted with plastic films; autoradiographic studies (Oppenheimer *et al.*, 1960) later indicated that the cells involved were confined to the connective tissue pocket surrounding the film. Increased amounts of metachromatic ground substance accompany fibroblastic proliferation in a number of circumstances (Taylor and Saunders, 1957) and this change is not an index of impending or actual malignant change.

One of the most striking features of the malignant changes induced by NTDQ is the way in which tiny irregular foci of abnormal fibroblasts and ground substance are organised into the discrete, well-circumscribed nodules which have been referred to as " early sarcomata ". Although there is convincing evidence that some of these nodules later invade the surrounding tissues, it is not established (a) whether the formation of early sarcomatous nodules is an essential intermediate stage before invasive lesions develop, or (b) how many of these early nodules become invasive. It is almost certain that not all foci of (presumptive) neoplastic cells develop into overt sarcomata, a view which is supported by the transplantation experiments of Stewart (1939) and by more recent observations by Oppenheimer *et al.* (1964). These workers showed that cellophane implanted into rats induced abnormal fibroblastic proliferation much earlier than implants of other plastics (4–5 weeks instead of 5–6 months); despite this premature activity, overt sarcomata induced by cellophane films did not appear significantly earlier and the eventual yield of macroscopic tumours was no greater.

One comment may be made on the relevance of these observations to the interpretation of carcinogenicity tests in the rat. If early sarcomatous changes are found, should they be included in any final assessment of carcinogenic potency? In the present circumstances, to do so would mean upgrading NTDQ from a weak carcinogen to a rather potent one. The need for realistic criteria in evaluating carcinogenicity tests in the rat has been cogently argued by Grasso and Golberg (1966); until much more is known of the incidence, distribution, natural history and significance of the early sarcomatous nodule, a proposal of this kind would be undoubtedly premature.

### SUMMARY

A rubber additive—polymerised N-nitroso-2,2,4-trimethyl-1,2-dihydroquinoline (NTDQ)—was administered for 20 weeks by the subcutaneous, intraperitoneal and oral routes to 120 young Sprague-Dawley rats. Local sarcomatous changes were induced in rats injected subcutaneously and (to a lesser extent) intraperitoneally; feeding of large doses of NTDQ was ineffective.

Some of the sarcomas which developed at injection sites were small lesions, only recognised at autopsy; in many animals, sarcomatous changes were not apparent until histological examination. These early sarcomatous lesions were studied in detail and three stages of development have been tentatively defined.

(1) Tiny foci of abnormal fibroblasts, associated with increased amounts of acid mucopolysaccharide ground substance, form between the pre-existing masses of multinucleate giant cells which are seen at sites of injection of NTDQ.

(2) These foci of abnormal fibroblasts enlarge and form circumscribed nodules, sharply demarcated from the surrounding tissues.

(3) Some of these nodules become irregular in outline, invade adjacent tissues, and are recognisable as macroscopic neoplasms.

Some of the changes evoked by NTDQ are reminiscent of the early neoplastic reactions associated with more powerful carcinogens such as the polycyclic hydrocarbons, macromolecular iron complexes, and certain plastics. The advantages of using a weak carcinogen such as NTDQ to trace the early development of induced sarcomata are discussed and some general implications of the present findings with regard to the interpretation of carcinogenicity testing are considered.

I am indebted to Dr. F. J. C. Roe for help with the preparation of this account; to Dr. Cuthbert Dukes for discussion of the histology; to Mrs. Joan Clack, Miss Diana Bishop and Mr. E. Woollard for technical assistance; and to Mr. K. G. Moreman and the staff of the Photographic Department, Chester Beatty Research Institute, for the photomicrographs.

This investigation has been supported by grants to the Chester Beatty Research Institute (Institute of Cancer Research: Royal Cancer Hospital) from the Medical Research Council and the British Empire Cancer Campaign for Research, and by the Public Health Service Research Grant No. CA-03188 from the National Cancer Institute, U.S. Public Health Service.

### REFERENCES

Baker, S. B. de C., Golberg, L., Martin, L. E. and Smith, J. F.—(1961) *J. Path. Bact.*, **82**, 453.

Boyland, E., Carter, R. L., Gorrod, J. W. and Roe, F. J. C.—(1968) *Eur. J. Cancer*, **4**, 233.

Carter, R. L.—(1969) *Br. J. Cancer* (in press).

Carter, R. L. and Roe, F. J. C.—(1969) *Fd Cosmet. Toxicol.* (in press).

Danishefsky, I., Oppenheimer, E. T., Willhite, M., Stout, A. P. and Fishman, M.—(1959) *Cancer Res.*, **19**, 1234.

Grasso, P. and Golberg, L.—(1966) *Fd Cosmet. Toxicol.*, **4**, 297.

Haddow, A. and Horning, E. S.—(1960) *J. natn. Cancer Inst.*, **24**, 109.

Oppenheimer, B. S., Oppenheimer, E. T., Stout, A. P., Willhite, M. and Danishefsky, I.—(1958) *Cancer, N.Y.*, **11**, 204.

Oppenheimer, E. T., Fishman, M., Stout, A. P., Willhite, M. and Danishefsky, I.—(1960) *Cancer Res.*, **20**, 654.

Oppenheimer, E. T., Willhite, M., Stout, A. P., Danishefsky, I. and Fishman, M.—(1964) *Cancer Res.*, **24**, 379.

Orr, J. W.—(1939) *J. Path. Bact.*, **49**, 157.

Pollard, M. and Kajima, M.—(1967) *J. natn. Cancer Inst.*, **39**, 135.

Pollard, M., Matsuzawa, T. and Salomon, J. C.—(1964) *J. natn. Cancer Inst.*, **33**, 99.

Richmond, H. G.—(1959) *Br. med. J.*, i, 947.

Roe, F. J. C. and Carter, R. L.—(1967) *Int. J. Cancer*, **2**, 370.

Roe, F. J. C., Dukes, C. E. and Mitchley, B. C. V.—(1967) *Biochem. Pharmac.*, **16**, 647.

Rondoni, P.—(1937) *Z. Krebsforsch.*, **47**, 59.

Stewart, H. L.—(1939) *Am. J. Path.*, **15**, 707.

Taylor, H. E. and Saunders, A. M.—(1957) *Am. J. Path.*, **33**, 525.

Vasiliev, J. M.—(1959) *J. natn. Cancer Inst.*, **23**, 441.

Vasiliev, J. M., Olshevskaja, L. V., Raikhlin, N. T. and Ivanova, O. J.—(1962) *J. natn. Cancer Inst.*, **28**, 515.